# Fatigue, barriers to physical activity and predictors for motivation to exercise in advanced Cancer patients

**DOI:** 10.1186/s12904-020-00542-z

**Published:** 2020-03-31

**Authors:** J. Frikkel, M. Götte, M. Beckmann, S. Kasper, J. Hense, M. Teufel, M. Schuler, M. Tewes

**Affiliations:** 1 West German Cancer Center, Department of Medical Oncology, University Hospital Essen, 45147 Essen, Germany; 2 Department of Pediatric Hematology/Oncology, Center for Child and Adolescent Medicine, University Hospital Essen, Essen, Germany; 3 Department of Psychosomatic Medicine and Psychotherapy, University of Duisburg-Essen, LVR-Klinikum Essen, 45147 Essen, Germany; 4 German Cancer Consortium (DKTK), Partner Site University Hospital Essen, 45147 Essen, Germany

**Keywords:** Physical activity, Advanced cancer patients, Barriers, Predictors, Motivation, Fatigue

## Abstract

**Background:**

In order to counteract fatigue, physical activity (PA) is recommended for all stages of cancer. However, only few advanced cancer patients (ACP) are physically active. Quantitative data with high numbers of ACP reporting barriers to PA are missing. This study aimed to identify barriers to PA in ACP with tiredness/weakness and investigate their motivation towards it.

**Methods:**

Outpatients with metastatic cancer receiving cancer care at a German Cancer Center reporting moderate/severe tiredness/weakness during self-assessment (MIDOS II) were enrolled. We assessed Fatigue-(FACF-F) and Depression (PHQ8) Scores, demographics, cancer-specific parameters, motivation for PA, physical, psychological and social barriers.

**Results:**

141 of 440 eligible patients (32.0%) with different diagnoses agreed to participate. Patients frequently reported *“I feel weakened due to my tumor therapy”* (*n* = 108; 76.6%), physical symptoms (tiredness, weakness, dyspnea, joint-problems, pain, nausea [*n =* 107; 75.9%]) and fatigue (*n* = 99; 70.2%) as barriers to PA. However, no significant group differences regarding these barriers were found between physically active and inactive patients. Social barriers were rarely chosen. Motivated patients were 5.6 times more likely to be physically active (*p <* 0.001), also motivation turned out to be the strongest predictor for a physically active behavior (β = 1.044; *p* = 0.005). Motivated attitude towards PA was predicted by *fatigue* (β = − 2.301; *p* = 0.008), *clinically relevant depression (*β = − 1.390, *p =* 0.039), *knowledge about PA and quality of life (QoL)* (β = 0.929; *p =* 0.002), *PA before diagnosis* (β = 0.688; *p =* 0.005 and *Interest in exercise program* (β = 0.635; *p =* 0.008).

**Conclusion:**

*“I feel weakened due to my tumor therapy”* is the most reported barrier to PA among both, physically and inactive patients. *Motivation for PA is the strongest predictor of performing PA. Interest in PA*, *knowledge about PA/QoL* and *PA before diagnosis* are main predictors of a motivated attitude*.* Absence/presence of social barriers did not associate with motivation, fatigue and depression proved to be a negative predictor. Programs including information, motivational counseling and individualized training should be offered for ACP to overcome barriers and reduce fatigue.

**Trial registration:**

German Register of Clinical Trials DRKS00012514, registration date: 30.5.2017.

## Background

Weakness and tiredness are the most frequent reported symptoms in patients with metastatic cancer (advanced cancer patients, ACP) [1]. Up to 91% of patients suffer from a level of weakness and tiredness that is more severe than the drowsiness experienced by healthy people and cannot be relieved by rest [2–4]. This symptom complex indicates the presence of cancer-related fatigue (CRF). Guidelines emphasize the significance of `treatable contributing factors` such as depression, anaemia, pain, cachexia, infection, and over dosage of sedatives, which should be clarified before the start of a treatment program for CRF [2, 5].

For treatment of CRF the National Comprehensive Cancer Network guidelines [2] recommend nonpharmacological interventions such as physical activity, physically based therapies and psychosocial interventions.

A large number of studies and meta-analyses confirmed that physical activity (PA) is an effective way to reduce CRF and to increase physical functioning during adjuvant cancer treatment [6–9]. Only few clinical trials demonstrated positive effects of PA in ACP with CRF [10–12]. However, to improve patients´ QoL and physical functioning [13], the American Cancer Society’s latest guidelines [14] recommend physical activity for patients with advanced tumor stage based on their personal physical abilities. But despite the scientific advice less than 30% of survivors manage to meet the current guidelines [15]. These findings are concordant to the results of few studies that examined PA behaviour in ACP with specific cancer types like prostate and lung cancer [16, 17].

Several studies investigated barriers to PA in cancer survivors. Recent studies described physical symptoms, comorbidities like cardiovascular, musculoskeletal and pulmonary diseases, weight gain and fatigue as main physical barriers. Depression, no motivation, fears, lack of knowledge about PA and QoL and no awareness of exercise program were mentioned as psychological barriers. Environmental and social related barriers were employment situation, access to facilities, bad weather and lack of time [18–23]. However, these findings cannot be transferred to the needs and requirements of ACP, since cancer survivors and ACP differ significantly regarding their life circumstances. Recent studies, mostly with an explorative and qualitative design, focusing on specific cancer types, detected (chemo) therapy related side effects such as tiredness, lack of energy and physical deconditioning as well as low motivation, bad weather and no access to facilities as main barriers to PA in ACP [24–27]. In addition to these studies further quantitative studies are needed. The objective of this study was to identify barriers to PA in a large cohort of ACP with mixed types of cancer that additionally suffer from the common symptom complex of tiredness and/or weakness. Furthermore, we aimed to determine predictors for motivation and PA itself.

## Methods

### Patient enrolment and study design

This prospective, non-interventional study was conducted in a high volume oncology outpatient unit of an university hospital in Germany and focused on outpatients with metastatic cancer. As a validated instrument to assess physical and cognitive symptoms in patients with advanced tumor disease the Minimal Documentation System (MIDOS II) [28] was used for patient selection. The MIDOS II includes 12 items in a 4-point Likert scale and can be regarded as the German version of the Edmonton System Assessment Scale (ESAS) [29]. Symptoms were routinely evaluated in all patients of the outpatient care unit mentioned above. Patients that indicated moderate to severe tiredness or weakness in the MIDOS II and had histologically confirmed metastatic cancer (UICC stage IV) were eligible to this study. Further inclusion criteria were age above 18 years, sufficient German language skills to answer the questionnaire and no severe cardiac or pulmonal impairment.

All cancer patients at the university hospital can participate in physiotherapeutic exercise programs by prescription. These include oncological training therapy, individual sessions or cancer sports groups for specific indications. Nevertheless, a comprehensive promotion of movement that goes beyond physiotherapy is still being developed and more information about the needs and wishes of patient groups with special needs is necessary.

All included participants received an information brochure and were asked to complete the paper-based questionnaire (see below) on the same day after signing the consent form.

### Questionnaire

The 64-item questionnaire included the Functional Assessment of Cancer Therapy Fatigue (FACT-F, [30–33]). Further contents of the questionnaire were the Patient Health Questionnaire depression scale (PHQ8, 8 items, [34–36]), demographics (6 items), psychological barriers (5 items), patients´ physical activity patterns (6 items), physical barriers (12 items) and social barriers (8 items).

For the collection of demographic data, patients were asked for nationality, marital status, living situation, number of children and their highest educational status.

Additionally, patients were asked for motivation for PA, whether they were physically active prior to their cancer diagnosis, interest in an exercise program and attitude about PA and QoL [18, 20, 37], in a 5-point Likert scale (0 = not at all, 1 = a little bit; 2 = somewhat; 3 = quite a bit; 4 = very much). Furthermore, patients could cross mark if they were physically active at the moment, how often (1–2 times, 2–3 times, more than 3 times per week) and with which intensity (light, moderate, severe) they worked out, if they had a training partner, if their partner was physically active and whether they were attending an exercise program.

To assess physical barriers in ACP [18, 20, 21], physical symptoms (weakness, pain, dyspnea, tiredness, vomiting/nausea and joint problems) could be marked. Patients were asked whether they felt weakened due to active systematic cancer therapy, whether they have had many hospital stays, their fear of damage from PA and their smoking status. Questions could be answered with “yes” or “no”. Additionally patients were asked to list their current medication.

To identify further barriers [18, 26] patients could mark the following statements if applicable: no local physiotherapist, no payed transport, missing prescription, lack of time, stressful daily life, too many other commitments, bad weather, PA is too expensive.

### Acquisition of data

Data about gender, tumor type, kind of cancer treatment, number of previous treatment lines, time since metastasis, performance status and comorbidities were extracted from electronic patient files at time of answered questionnaire. For calculation of body mass index weight and height measured up to 21 days before date of answered questionnaire was used.

### Statistical analysis

Percentage, frequencies, median, mean values and standard deviations were calculated for descriptive analysis by SPSS (Version 23). To measure the strength of relation between the barriers to PA of physically active and inactive participants we calculated the relative risk (RR) and the 95% confidential interval for each barrier. Depending on the sample sizes and scale level parametric (*T* test for independent variables) and non-parametric (Fisher’s exact Tests, χ2-Test, Mann- Whitney-*U* Tests) tests were used for group comparisons. For analyses and the calculation of relative risk the answers “not at all”/“a little bit” and “somewhat”/ “quite a bit”/ “very much” of the 5-point Likert scale were summarized. A series of binary multiple logistic regression analysis was performed to quantify the variables that are able to predict a physically active behavior (defined as being physically active at least once a week) of PA and their motivation for PA. Patient reported variables which turned out to distinguish between physically active or inactive patients were included in the regression model to quantify the relative contribution of these variables in prediction of the criterion variable to detect predictors for motivation for PA. To measure the outcome of the binary dependent variable “Motivation for PA” patients´ answers “not at all”/“a little bit” and “somewhat” of the question “How motivated are you to exercise?” were summarized as “no” and “quite a bit”/ “very much” were summarized as “yes”. Tests for multicollinearity turned out to be negative, so the included variables are not highly correlated. We considered *p* < .05 to indicate statistical significance.

## Results

**Fig. 1:**
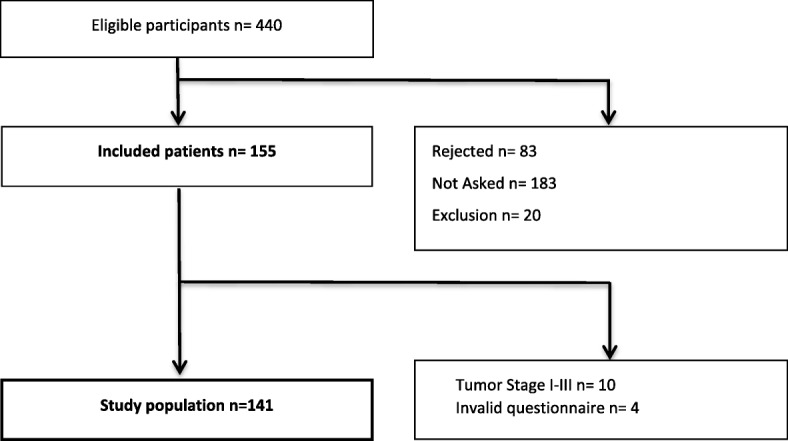
Patient Recruitment

During the routinely performed patient reported outcome measures between May 2017 and August 2018, 1361 questionnaires were completed. Moderate to severe tiredness and/or weakness were detected in 725 (53.3%) MIDOS II questionnaires answered by 440 patients. Finally, 141 participants have been enrolled in the study **(**Fig. 1**).**

### Characteristics of patients

Details of the participants’ characteristics are presented in Table 1. Thirty-one patients (22.0%) were physically active at least once a week. Overall, patients had a high performance status (ECOG 0: 48.2%, *n =* 68; ECOG 1: 35.5%; *n =* 50) and active patients had a significantly better performance status (ECOG 0: 64.5%, *n* = 20, *p* = 0.04). Forty patients (28.4%) had an elevated c-reactive protein (> 20 mg/l). Most frequent comorbidities were cardiovascular diseases (*n =* 81; 57.4%), anemia (*n* = 39; 27.7%) and musculoskeletal disorders (*n* = 31; 22.0%). Eight patients (5.7%) were underweight (body mass index < 18.5 kg/m^2^). The group of “physically active patients” showed significantly less comorbidities than inactive patients (*p =* 0.008).

**Table 1:** Patients’ characteristics

Characteristic	All *(n = 141)*	Physically inactive ^a^*(n = 110)*	Physically active ^a^*(n = 31)*	Group differences (*p value*)
Mean age (SD; range), years	60 (±11; 26–83)	61 (±11; 26–83)	59 (±11; 35–80)	
Sex (%)				
Female	85 (60.3)	64 (58.2)	21 (67.7)	
Male	56 (39.7)	46 (41.8)	10 (32.3)	
Tumour type (%)				
Gastrointestinal Cancer	44 (31.2)	36 (32.7)	8 (25.8)	
Lung Cancer	40 (28.4)	32 (29.1)	8 (25.8)	
Breast Cancer	28 (19.9)	17 (15.5)	11 (35.5)	
Sarcoma	11 (7.8)	11 (10.0)	0 (0)	
Others ^b^	11 (7.8)	8 (7.3)	3 (9.7)	
Head and Neck Cancer	7 (5.0)	6 (5.4)	1 (3.2)	
Previous adjuvant chemotherapy (%)	43 (30.5)	31 (28.2)	12 (38.7)	
Previous palliative chemotherapy (%)	84 (59.6)	68 (61.2)	16 (51.2)	
Number of lines, mean (SD; range)	1.77 (±1.1; 1–7)	1.67 (±1.1; 1–7)	2.25 (±1.3; 1–5)	
Duration since metastatic cancer, months, mean (SD; range)	22.7 (±26.0; 0–147)	19.5 (± 22.2; 0–147)	34.0 (±34.6; 2–137)	***
Current therapy ^c^ (%)				
Chemotherapy	91 (64.5)	74 (67.3)	17 (54.8)	
Immunotherapy	28 (19.9)	22 (20.0)	6 (19.4)	
Antibody therapy	8 (6.4)	5 (4.5)	3 (9.7)	
Combined w/ Hormonal treatment	3 (2.1)	1 (0.9)	2 (6.5)	
Monotherapy	5 (3.5)	4 (3.6)	1 (3.2)	
Targeted therapy	11 (7.8)	6 (5.5)	5 (16.1)	
Combined w/ Hormonal treatment	7 (5.0)	2 (1.8)	5 (16.1)	
Monotherapy	4 (2.8)	4 (3.6)	0 (0)	
Hormonal treatment	3 (2.1)	3 (2.7)	0 (0)	
ECOG-status (%)				
0	68 (48.2)	48 (43.6)	20 (64.5)	
I	50 (35.5)	39 (35.5)	11 (35.5)	
II	13 (9.2)	13 (11.8)	0 (0)	
III	1 (0.7)	1 (0.9)	0 (0)	
Missing	9 (6.4)	9 (8.2)	0 (0)	
Comorbidities (%)				
Cardiovascular disease	81 (57.4)	68 (61.8)	13 (41.9)	
Anemia ^d^	39 (27.7)	32 (29.1)	7 (22.6)	
Musculoskeletal disorder ^e^	31 (22.0)	26 (23.6)	5 (16.1)	
Thyroid gland disease	29 (20.6)	26 (23.6)	3 (9.7)	
Pulmonary disease	27 (19.1)	24 (21.2)	3 (9.7)	
Diabetes mellitus	24 (17.0)	22 (20.0)	2 (6.5)	
Psychiatric disease	12 (8.5)	9 (8.2)	3 (9.7)	
Polyneuropathy	5 (3.5)	3 (2.7)	2 (6.5)	
Number of comorbidities, mean (SD, range)	2.51 (±2.0; 0–10)	2.75 (± 2.0; 0–10)	1.68 (± 1.8; 0–7)	***

Abbreviations: *ECOG* Eastern Co-operative Oncology Group performance index, *SD* standard deviation

*** significant group differences with *p* < 0.05

^a^ Physically inactive = no activity at all; physically active = at least 1 time/week with low Intensity

^b^ Genitourinary Cancer (*n* = 4), other Gynaecologic Cancers (n = 2), CUP (n = 1), Glioblastoma (n = 1), Others (n = 3)

^c^ Last therapy before answering the Questionnaire

^d^ A haemoglobin value < 10.0 mg/dl was defined as “Anemia”

^e^ Present musculoskeletal disorders were Arthrosis, Osteoporosis, Joint infection, Bechterews disease, Chronical Pain Syndrome, Herniated Disc, Rheumatoid Arthritis

The following potentially `treatable contributing factors` were detected: 40 patients (28.4%) had a clinically significant depressive disorder (PHQ8 > 10), 39 patients (27.7%) suffered from anemia (haemoglobin < 10 g/dL), intake of sedatives was detected in 13 patients (9.2%) and 8 patients (5.7%) had cachexia (body mass index < 18.5 kg/m^2^) and severe pain (self-assessment).

### Barriers to physical activity

**Fig. 2:**
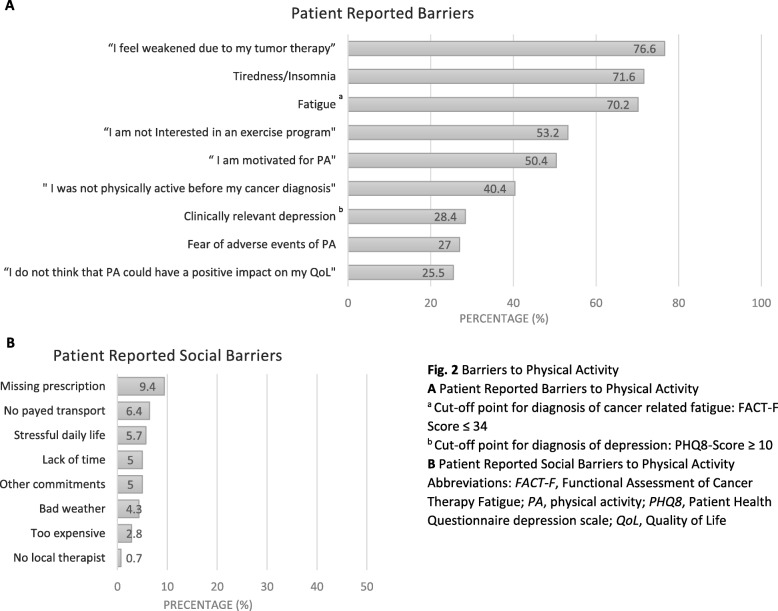
Barriers to Physical Activity **a** Patient Reported Barriers to Physical Activity ^a^ Cut-off point for diagnosis of cancer related fatigue: FACT-F Score ≤ 34 ^b^ Cut-off point for diagnosis of depression: PHQ8-Score ≥ 10 **b** Patient Reported Social Barriers to Physical Activity Abbreviations: *FACT-F*, Functional Assessment of Cancer Therapy Fatigue; *PA*, physical activity; *PHQ8*, Patient Health Questionnaire depression scale; *QoL*, Quality of Life

Figure 2 demonstrates the main barriers to PA. Most common patient reported barriers (Fig. 2a) were *“I feel weakened due to my tumor therapy”* (*n* = 108; 76.6%), tiredness/insomnia (*n* = 101; 71.6%) and a pathological FACT-F score (< 34) (*n* = 99; 70.2%) indicating the presence of CRF [33]. Patients marked the following physical barriers: weakness 89 patients (63.1%), dyspnea: 49 patients (34.8%), joint problems: 44 patients (31.2%), pain: 43 patients (30.5%), nausea/vomiting: 16 patients (11.3%). Frequent potential barriers mentioned in the patient files were comorbidities (*n* = 120; 85.0%), 92 patients (65.2%) suffered from more than two comorbidities. Approximately half of the patients were overweight (BMI > 25 kg/m^2^; *n* = 60; 42.6%, mean 25.6 ± 6.0 range 15.0–50.2). On the average, participants cross-marked 0.39 (± 0.85; 0–4) of the eight items referring to social barriers. Details concerning social barriers are presented in Fig. 2c.

### Group comparison of physically active and inactive patients

Group comparisons in barriers to PA between physically active and inactive patients as well as calculations of relative risk (RR) are shown in Table 2.

**Table 2:** Group comparison of physically active and inactive patients

	All (%) *(n = 141)*	Physically Inactive (%)^a^*(n = 110)*	Physically active (%)^a^*(n = 31)*	RR (95% CI)	*P-*value
Patient Reported Barriers					
“*I feel weakened due to my tumor therapy”*	108 (76.6)	84 (76.4)	24 (77.4)	1.09 (0.52–2.27)	0.807
Missing	4 (2.8)	4 (3.6)			
Tiredness/Insomnia ^b^	101 (71.6)	78 (70.9)	23 (74.2)	1.18 (0.48–2.91)	0.524
*“I think that PA could have a positive impact on my QoL”*^d^	102 (72.3)	75 (68.2)	27 (87.1)	2.38 (0.90–6.4)	0.066
Missing	3 (2.1)	3 (2.7)			
**Fatigue**^b^	**99 (70.2)**	78 (71.6)	21 (67.7)	1.12 (0.58–2.17)	0.825
**“I am Interested in an exercise program”**^**d**^	**75 (53.2)**	**51 (46.4)**	**24 (77.4)**	**2.79 (1.29–6.03)**	**0.007**
**Missing**	**5 (3.5)**	**5 (4.5)**			
**“I was physically active before my cancer diagnosis”**^**d**^	**84 (59.6)**	**60 (54.6))**	**24 (77.4)**	**2.25 (1.04–4.85)**	**0.037**
Missing	2 (1.4)	2 (1.8)			
“***I am motivated for PA*”**^***d***^	**66 (46.8**)	**40 (36.4)**	**26 (83.9)**	**5.56 (2.28–13.71)**	**< 0.001**
Missing	4 (2.8)	4 (3.6)			
Clinically significant depression ^e^	40 (28.4)	34 (30.9)	6 (19.4)	1.65 (0.73–3.72)	0.262
Fear of adverse events of PA	38 (26.9)	33 (30.0)	5 (16.1)	1.98 (0.82–4.79)	0.166
Missing	7 (5.0)	6 (5.5)	1 (3.2)		
Patient Related Barriers					
**≥ 2 Comorbidities**^**c**^	**92 (65.2)**	**79 (71.8)**	**13 (41.9)**	**1.71 (1.11–2.63)**	**0.003**
Overweight	60 (42.6)	49 (44.5)	11 (35.5)	1.01 (0.42–2.54)	1.000
Missing	15 (10.6)	6 (5.4)	9 (29.0)		
**ECOG < 2**	**118 (83.7)**	**87 (79.1)**	**31 (75.6)**	**1.36 (1.22–1.51)**	**0.04**
Missing	9 (6.4)	9 (8.2)			
Age > 65 years	44 (31.2)	36 (32.7)	8 (25.8)	1.40 (0.57–3.43)	0.518
**Tumor type (breast cancer vs. others)**	**28 (19.9)**	**17 (15.4)**	**11 (35.5)**	**3.0 (1.22–7.40)**	**0.02**

Abbreviations: *CI* confidence interval, *CTx* cancer therapy, *ECOG* Eastern Co-operative Oncology Group performance index. *FACT-F* Functional Assessment of Cancer Therapy Fatigue, *PA* physical activity, *PHQ8* Patient Health Questionnaire depression scale, *QoL* Quality of Life, *RR* relative risk

^a^ Physically inactive = no physical activity at all; Physically active = at least 1 time/week with low Intensity

^b^ FACT-F Score ≤ 34: cut-off point for diagnosis of cancer related fatigue [32]

^c^ Comorbidities were classified in the following categories: Cardiovascular disease, Anemia, Orthopaedic disease, Thyroid gland disease, Pulmonary disease, Diabetes mellitus, Psychiatric disease, Polyneuropathy

^d^ The 5-point scale that was used during the survey was divided into two sections: ‘not at all`/`a little bit` were matched with `low motivation` or `no`; `somewhat`/ `quite a bit`/ `very much’ were matched with `high motivation’ or `Yes`

^e^ A PHQ8-Score ≥ 10 points to clinically relevant depression [35]

#### Patient reported barriers

No significant differences concerning the statement *“I feel weakened due to my tumor therapy*”, any specific physical barriers (tiredness/insomnia, weakness, dyspnea, joint problems, pain, nausea/vomiting) or the prevalence of CRF were found between physically active and inactive patients. These two subgroups significantly varied in reporting *“I am motivated for PA”* [RR 5.56; 95%CI 2.28, 13.71; *p <* 0.001]*, “I am interested in an exercise program*” [RR 2.79; 95%CI 1.29–6.03; *p =* 0.07] and “I was physically active before my cancer diagnosis” [RR 2.25; 95%CI 1.04–4.85; *p =* 0.037].

#### Patient related barriers

With respect to age-group (> 65 years), the frequency of receiving chemotherapy or being overweight (body mass index> 25 kg / m2) both subgroups were equal but differed by the proportion of patients with good performance status (ECOG< 2) [RR 1.36; 95%CI 1.22–1.51; *p =* 0.04] and those having less than two comorbidities [RR 1.71 95%CI 1.11–2.83; *p =* 0.003].

#### Patient reported social barriers

Analyses of social barriers showed no significant variations between physically active and inactive respondents. In addition, the subgroups showed no differences in their demographic data regarding educational attainment (no/lower Degree versus intermediate/high degree) [RR 1.9; 95%CI 0.60–2.33; *p =* 0.674] and employment status (employed/not employed) [RR 1.27; 95%CI 0.67–2.41; *p =* 0.50].

#### Breast cancer patients

Patients with breast cancer were 3.0 times more likely to be physically active compared to all other tumor types [RR 3.0; 95%CI 1.22–7.40; *p =* 0.02]. A further subgroup analysis showed that breast cancer patients had a significantly longer duration since metastasis compared to other tumor types (40.0 ± 35.0 months, range 5–137, *p* < 0,001). Mean duration since metastatic cancer was significantly higher in physically active patients (34.0 ± 6.0 months, range 2–137, *p* = 0.025) than in inactive patients (19.5 ± 22.2 months, range 0–147). Further group comparisons regarding the participants´ gender did not detect any differences in PA between male and female patients [RR 0.65; 95% CI 0.28–1.52; *p* = 0.407].

### Predictors of physically active behavior and motivation for physical activity

The binary regression model conducted to identify predictors for performing PA was significant and showed an explained variation of 48.3% (*R*^*2*^ = 0.483, *p* < 0.001). The following variables were included in the model to explain physically active behavior: Age, sex, ECOG-Performance status ≥2, number of comorbidities, tumor type (breast cancer vs. others), duration since metastatic cancer (months), Motivation for PA, “I was physically active before my cancer diagnosis”, “I am interested in an exercise program” and “I think PA could have a positive impact on my QoL”. Of these variables only *“Motivation”* (β = 1.044 *p* = 0.005) and *“Duration since metastatic cancer, months*” (β = 0.026, *p* = 0.017) were significant (Table 3). The following regression analysis was conducted to determine predictors of motivation for PA. The model was significant with an explained variation of 69.2%, which points to a strong goodness-of-fit (f = 0.86) according to Cohen [38]. The model was adjusted for the following variables: Age, sex, receiving chemotherapy, active systemic cancer treatment, previous palliative chemotherapy lines, ECOG-Performance status ≥2, number of comorbidities and tumor type (breast cancer vs. others), pathological FACT-F Score, clinically relevant depression, “I was physically active before my cancer diagnosis”, “I am interested in an exercise program” and “I think PA could have a positive impact on my QoL”. Significant predictors of motivation for PA were “pathological FACT-F Score” (ß = − 2.301; *p* = 0.008), “clinically relevant depression” (ß = − 1.390, *p =* 0.039), knowledge *about PA and QoL* (ß = 0.929; *p* = 0.002), *PA-lifestyle before cancer diagnosis* (ß = 0.688; *p* = 0.005) and *interest in exercise program* (ß = 0.635; p = 0.008). Table 3 depicts the predictors of PA and motivation for PA.

**Table 3:** Predictors of Physically Active Behavior and Motivation for Physical activity

Dependent variable Significant predictors beta Wald *p*				
Physically Active Behavior (R^2^ = 0.483; f = 0.97)^a^	1. *Motivation for PA*	1.044	7.76	0.005
	2. *Duration since metastatic cancer (months)*	0.026	5.68	0.017
Independent variables in the regression models: age, sex, ECOG ≥2, number of comorbidities, tumor type (breast cancer vs. others), PA before cancer diagnosis, Interest in exercise program and knowledge about PA and QoL Quality of regression model: *p* < 0.001; χ2 = 48.4				
Motivation for PA (R^2^ = 0.685; f = 1.47)^a^	1. *Fatigue b*	-2.301	7.03	.008
	2. *Clinically relevant depression c*	-1.390	4.25	.039
	3. *Knowledge about PA and QoL*	.929	9.20	.002
	4. *PA before cancer diagnosis*	.688	7.89	.005
	5. *Interest in exercise program*	.635	6.97	.008
Independent variables in the regression models: age, sex, ECOG-Performance status ≥2, number of comorbidities, tumor type (breast cancer vs. others) Quality of regression model: *p* < 0.001; χ2 = 87.9				

Abbreviations: *ECOG* Eastern Co-operative Oncology Group performance index, *PA* physical activity, *QoL* quality of life

^a^ R^2^ = Explained variance; f = Effect size according to Cohen;

^b^ FACT-F Score ≤ 34: cut-off point for diagnosis of cancer related fatigue [32]

^c^ PHQ8-Score ≥ 10 points to clinically relevant depression [35]

## Discussion

Clinical practice guidelines [2, 5] recommend PA for patients suffering from fatigue. Despite this advice, only few patients manage to be physically active during their cancer treatment. Consistent with our findings some authors have demonstrated that approximately one third of ACP manage to meet the current scientific advice [16, 17, 39]. The purpose of this study was to identify barriers to PA in ACP with tiredness and/ or weakness and investigate their motivation towards it.

Our analysis found that the statement *“I feel weakened due to my tumor therapy”* was the most common patient-reported barrier for PA, followed by several *physical symptoms* (mainly tiredness/insomnia and weakness) and a pathologic score of FACT-F as an indicator for a clinically relevant level *fatigue.* Numerous previous studies also described these variables as barriers to PA in cancer patients [19, 24, 26]. Interestingly, the observed subgroups of physically active and inactive patients showed no differences regarding the presence of these physical barriers [25, 27]. The finding might show us, that patients find different strategies to overcome these frequently reported barrier (see Table 2). This gives us a hint on how different patients deal with their individual barriers towards physical activity and underlines the importance of developing strategies of reducing the negative impact of physical symptoms like fatigue or weakness due to cancer therapy on patients´ behavior. This interesting question could be evaluated in a qualitative analysis or an investigation in a mixed-methods design. Our data suggests that the improvement of patients´ motivation for PA might provide a helpful opportunity.

Only 47% of participants claimed to be interested in an exercise program*.* These patients were significantly more likely to be physically active. A pilot study conducted by Lowe et al. [40] in 2013 also showed low interest of ACP in participating and completing an exercise program. Only 77 of 524 ACP agreed to join a 6-week home based workout program. However, this population should be motivated to start and stick to PA. Therefore, our study investigated potential predictors of motivation.

Physically active and inactive patients differed significantly in PA before cancer diagnosis. Patients exercising before their diagnosis were two times more likely to be physically active during treatment. These results were in concordance with several studies. Eng et al. [23] examined PA behavior, perceptions and perceived barriers to PA in a study cohort of 1003 cancer survivors. In this study, patients with a sedentary lifestyle before diagnosis rarely improved their activity level after diagnosis. However, this group was more likely to increase PA levels when perceiving that PA improves their QoL. Despite these findings, only 13% of cancer survivors received PA counseling by health care providers. Although there are obvious recommendations for PA in ACP, Hardcastle et al. [41] demonstrated, that only 46% of oncologists promote PA in their patients. In contrast to these results, most patients of our survey were educated about the positive effects of PA on QoL which could be explained be the monocentric conduction of our study. Our oncologic outpatient unit has an attached importance to early integration of supportive care such as exercise therapy.

Lack of motivation is already a well-known barrier to PA in cancer patients [18, 26]. In our study we measured motivation for PA in ACP and compared the results in physically inactive and active patients. Patients that claimed to be motivated for PA were 5.6 times more likely to be physically active at least once a week. Within the scope of a small explorative study with 5 ACP, Mas et al. [25] suggested that psychological factors might be determining whether patients are physically active or not. Physical factors on the contrary might have influence on intensity, regularity and the duration of PA. Clark et al. [27] demonstrated that the feeling of self-efficacy in ACP was not significantly associated with the physical condition of a patient. According to Clark et al. [27] clinicians should be aware that the presence of physical symptoms do not implicate a lack of motivation and confidence for PA in ACP. Our findings support these positions: physically active and inactive patients rarely differed in the presence of physical barriers, whereas our analysis of several psychological factors, especially motivation for PA showed significant differences.

Our group comparisons showed, that patients with breast cancer were 3.0 times more likely to be physically active compared to patients with other tumor types. Interestingly, there is a lot of evidence for the positive impact of PA on the QoL in patients dealing with breast cancer [42]. Several systematic reviews [43–45] summarized the improvement of fatigue, self-reported physical functioning and health-related quality of life due to PA in this patient group. Furthermore, in our study patients with breast cancer had a significantly longer duration of metastatic disease. This variable turned out to be a positive predictor of a physically active behavior. Nevertheless these findings cannot be generalized on the basis of such small subgroup-analyses. Further research on this topic is required.

The sector of social barriers was rarely chosen by our cohort. These results differ from previously recorded data, where `lack of time`, ´bad weather` and `lack of suitable facilities` were frequently mentioned barriers to PA in cancer survivors [18–20]. Hence, only one study identified social barriers in ACP [26]. Possibly cancer survivors and ACP have different requirements regarding their social environment, since these groups differ significantly in their life situation. Semi-structured interviews, open questions and a triangulation in a mixed-methods design might bring up extensive results in social barriers of ACP. Still, further research in the social requirements for PA of ACP is needed. Additionally, the access to supportive care institutions such as physiotherapy and rehabilitation programs may depend on the countries´ health care systems. Our monocentric study was located in an urbanized area of Germany with a high density of physiotherapeutic teams. This also might be a reason for the deviation of results. Furthermore, ACP seem to have different needs regarding the implication of PA: our study clearly demonstrates that physical barriers are more important in this cohort.

In our study, a pathological score of FACFT-F – as an indicator of *fatigue-* turned out to be the strongest negative predictor of *motivation for PA*. This important result is in line with several studies investigating barriers to PA in ACP [25, 26] and cancer survivors [19, 21, 23]. These authors described fatigue as a main barrier of PA. In addition to these studies, our analysis demonstrates *clinically relevant depression* as an important negative predictor of motivation for PA, although depression was present in only approximately one third of our cohort. This prevalence is in concordance with the findings of Walker et al. [46]. Other studies presented depression as a barrier to PA in cancer survivors with colorectal and breast cancer [19, 47]. To our best knowledge depression in ACP as a barrier to PA is not well described and further investigations are needed. Furthermore, regression analyses identified *interest in exercise program*, *knowledge about PA and QoL* or *PA before cancer diagnosis* as significant positive predictors for a motivated attitude towards PA*.* In this respect, especially patients with PA before cancer diagnosis should be encouraged to be physically active. Although our group comparisons showed, that patients with good performance status, less comorbidities and patients with breast cancer were significantly more likely to be physically active, these factors turned out to be independent variables in the regression models of PA and motivation for PA. The previous and intrinsic motivation as well as the education about PA during cancer treatment may help our patients to overcome their physical burdens and get physically active during their cancer treatment.

Our findings should be interpreted in the light of several limitations. This monocentric study was conducted in an outpatient care of a large oncologic center in Germany. Therefore, our study population might not be representative regarding different diagnoses among our cohort. Cancer cachexia might be another important barrier for PA in ACP. In our study, we only measured the patients´ body mass index. To define the multifactorial syndrome of cancer cachexia more aspects (e.g. percent weight loss [48]) should be considered [49]. Another limitation of our study is the small sample size of physically active patients. This prohibits the generalization of our results. Still, only few ACP are physically active and our study aimed at finding reasons for this circumstance.

## Conclusion

Only few ACP with tiredness and/ or weakness manage to be physically active. Most patients reported *“I feel weakened due to my cancer therapy*”, physical *symptoms* (mainly tiredness/insomnia and weakness) and *fatigue* as barriers for PA. Interestingly there were no significant group differences in these mentioned barriers between physically active and inactive patients. Patients with motivation for PA seem to be more likely to overcome their reported barriers, since this subgroup is 5.6 times more likely to be physically active. Since motivation for PA turned out to be the strongest predictor for a physically active behavior, the identified positive predictors for motivation itself (*interest in exercise program, knowledge about the positive impact of PA on QoL and PA before cancer diagnosis)* emphasize the need for supportive health care providers who help patients counteract the negative impact of fatigue and depression. The group comparisons also support the notion that attractive exercise programs are critical to increase patients’ interest in PA. Interdisciplinary programs including psychoeducation about PA and QoL, motivational counseling and feasible exercise programs adjusted to the individuals´ needs and abilities may strengthen patients´ motivation and help to overcome barriers for PA*.* Further studies are needed to investigate successful approaches to increase motivation for PA in ACP.

## Data Availability

The datasets generated and/or analysed during the current study are not publicly available due to ongoing acquisition of follow-up data but are available from the corresponding author on reasonable request.

## Abbreviations

ACP: Advanced cancer patients
CI: Confidence interval
CRF: Cancer related fatigue
ECOG: Eastern cooperative oncology group
FACT-F: Functional Assessment of Cancer Therapy- Fatigue
FACT-G: Functional Assessment of Cancer Therapy- General
Fig: Figure
MIDOS: New Minimal Documentation System
PA: Physical activity
PHQ8: Patient Health Questionnaire depression scale
QoL: Quality of life
RR: Relative risk
UICC: Union for International Cancer Control
